# Expression and prognosis analyses of CASP1 in acute myeloid leukemia

**DOI:** 10.18632/aging.203028

**Published:** 2021-05-17

**Authors:** Jing Liu, Minyi Zhao, Xiaohui Feng, Yunxin Zeng, Dongjun Lin

**Affiliations:** 1 The Seventh Affiliated Hospital of Sun Yat-Sen University, Shenzhen 518107, Guangdong, People’s Republic of China

**Keywords:** CASP1, expression, apoptosis, prognosis, AML

## Abstract

Caspase1 (CASP1) is a gene that encodes multiple proteins related to cell death. Nevertheless, the function of CASP1 in the pathogenesis of AML is still unclear. In the present study, a detailed analysis of cancer versus normal samples was performed to explore the relationship between CASP1 and leukemia. We used sequencing data from multiple cancer gene databases to analyze the gene expression and regulatory network of CASP1 in leukemia. We discovered that mRNA expression levels of CASP1 are increased in leukemia cell lines, especially in acute myelocytic leukemia (AML). Then, we verified the mRNA expression of CASP1 in AML clinical samples and observed significantly higher expression of CASP1 in relapsed AML patients. High CASP1 expression was associated with poor prognosis and CASP1 inhibition could impair the proliferation of AML cells. Related functional network identification suggests that CASP1 regulates apoptosis, immune and inflammatory response via pathways involving LYN, LCK, and the E2F family. These findings suggest that CASP1 probably contributes to the pathogenesis, and identify CASP1 as a factor for predicting the prognosis and as a therapeutic target of AML patients.

## INTRODUCTION

Leukemia is the most common cause of hematologic malignancies in which immature precursors proliferate and accumulate in bone marrow and other hematopoietic tissues due to abnormal proliferation and differentiation [1]. Survival rates and prognosis are poor due to a high rate of refractory recurrence. The therapeutic effects of existing drugs are poor owing to the complex molecular mechanism of leukemia and chemoresistance [1, 2]. In addition, a lack of clear drug targets for leukemia is a critical factor in its treatment. Cascade activation of caspases is closely related to multiple cell death processes.

As a member of caspases family, CASP1 was first isolated from the human monocyte cell line THP1. It has been regarded as a key enzyme of the apoptotic pathway [3, 4]. CASP1 is involved in proteolysis, protein auto processing, apoptosis, and several important signaling pathways [5–11]. Inflammasome plays a crucial role in the process of chronic inflammation and carcinogenesis [12]. CASP1, a key component of the inflammasome, had been reported to have a profound impact on tumors formation and progression in multiple human cancers [13, 14]. CASP1 decreased significantly in prostate cancer [15] and ovarian cancer, indicating that down-regulation of CASP1 may play an important role in the carcinogenesis and progression of tumors [16]. However, it has been reported that genetic variants common in genes associated with apoptosis and immune regulation are associated with risk of chronic lymphocytic leukemia (CLL) [17]. Meanwhile, CASP1 and its activator NLRP3 are the core component of the inflammasome, and its overexpression in ALL (Acute lymphocytic leukemia) leads to glucocorticoid resistance [18]. Although the role of CASP1 in carcinogenesis is controversial, it may be a prospective indicator for leukemia prevention and treatment.

Here, we performed data mining on the expression and prognosis of CASP1 in AML patients from the Cancer Genome Atlas (TCGA) and multiple cancer databases. Then, we verified the mRNA expression of CASP1 in AML cell lines and clinical samples. Through multidimensional analysis, we assessed the functional network and prognostic value associated with CASP1 in AML. Our results could potentially reveal new targets for AML diagnosis and treatment.

## RESULTS

### Transcriptional expression of CASP1 in leukemia

**Figure 1:**
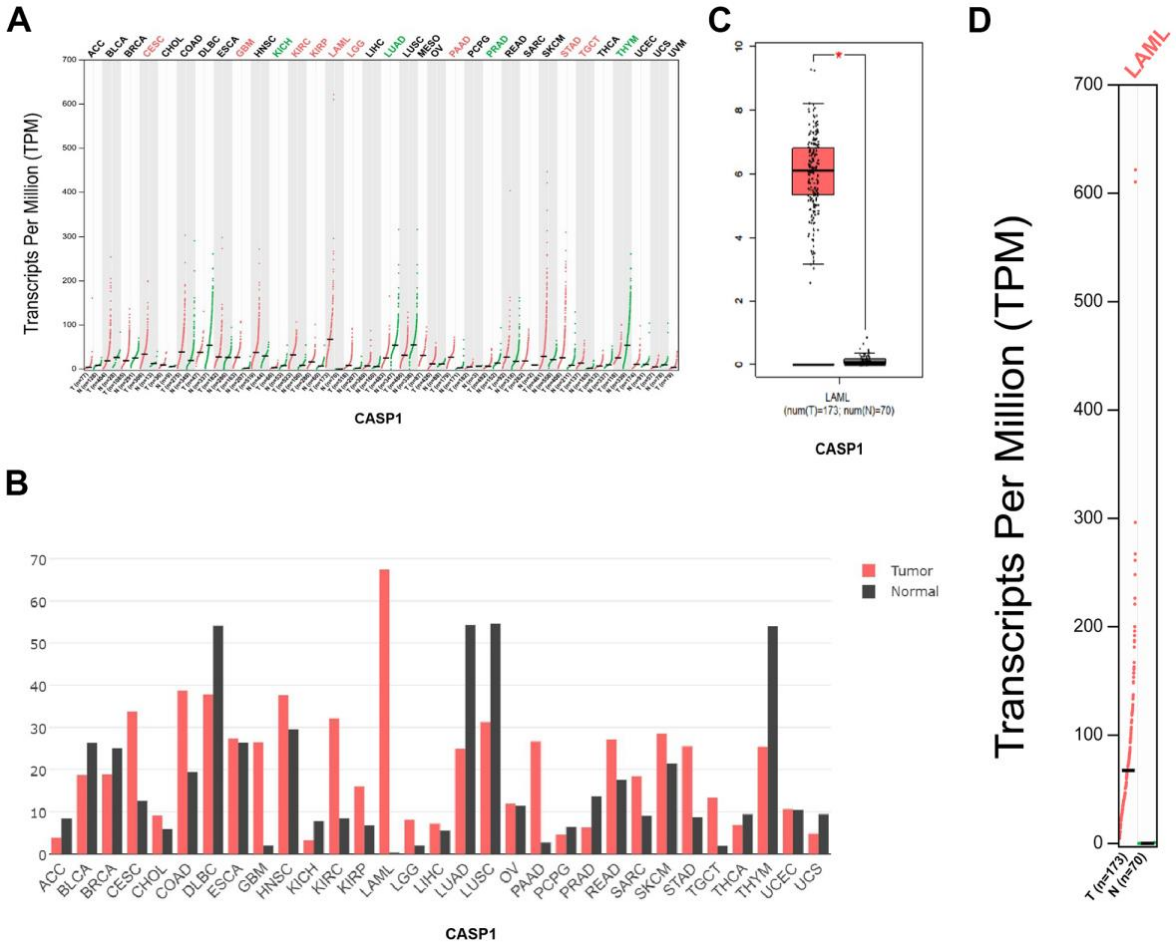
**CASP1 expression in leukemia (GEPIA).** (**A**, **B**) CASP1 expression in multiple cancers. (**C**, **D**) CASP1 expression in AML.

We performed a retrieval of CASP1 mRNA expression levels in a variety of cancers by using the GEPIA database. We discovered that mRNA expression levels of CASP1 in patients with leukemia were upregulated compared with normal samples (Figure 1A–1D).

**Figure 2:**
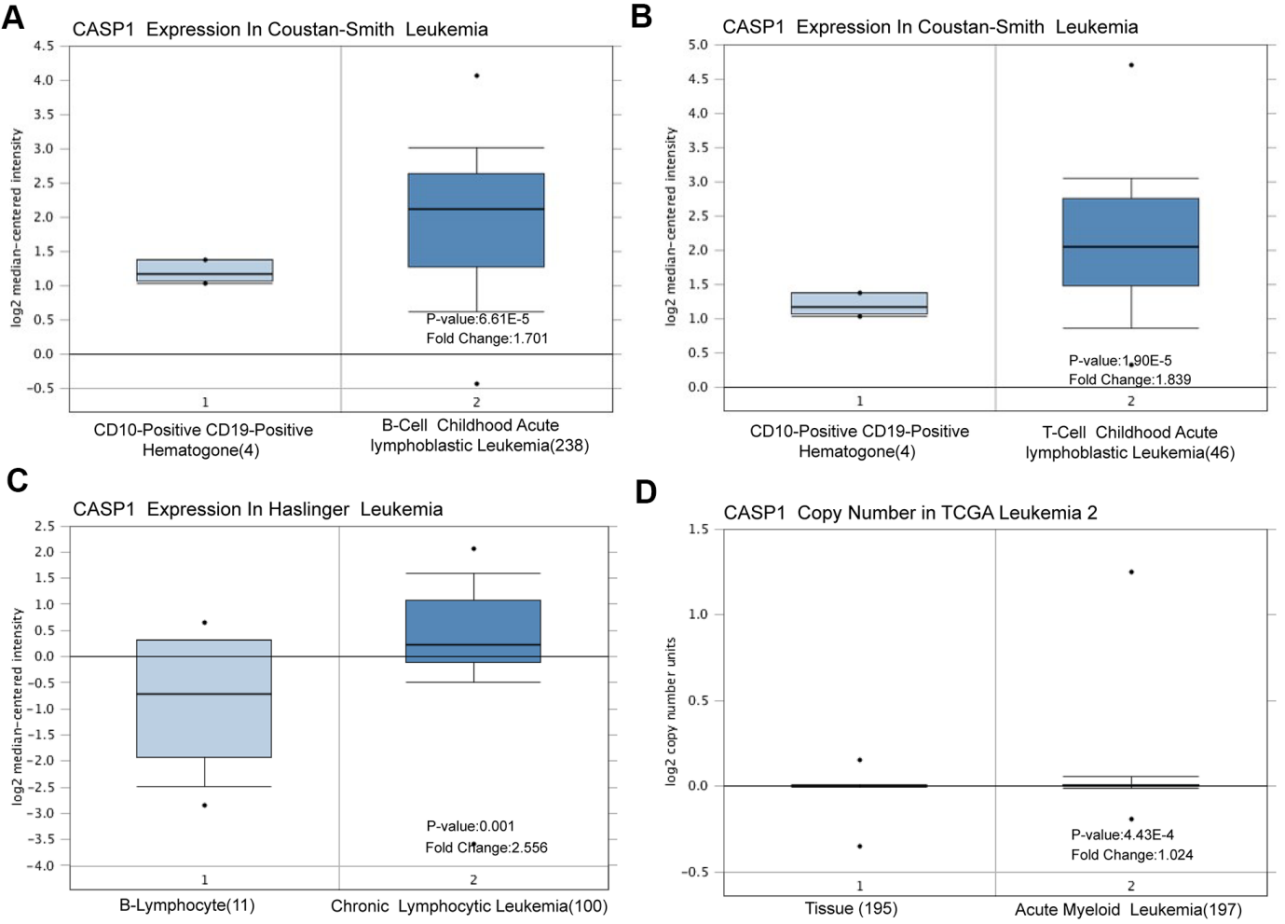
**CASP1 transcription in leukemia (Oncomine).** CASP1 mRNA expression and DNA copy number were highly in leukemia than in normal blood samples. (**A**–**C**) CASP1 mRNA expression levels were shown by box plot in Constant-Smith leukemia, and Haslinger leukemia respectively. (**D**) CASP1 copy number was shown by box plot in The Cancer Genome Atlas (TCGA) Leukemia 2 datasets.

**Figure 3:**
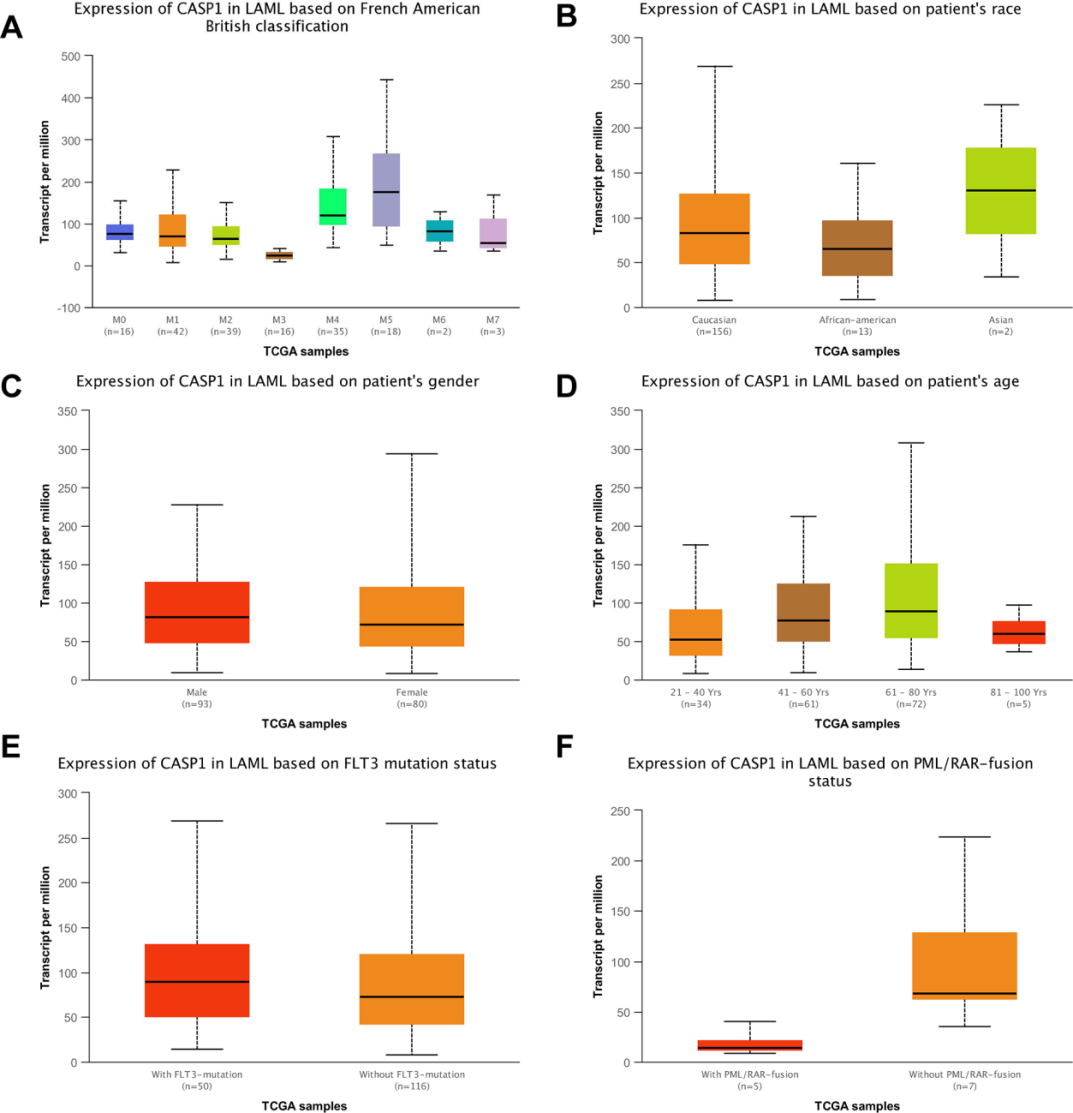
**CASP1 transcription in the leukemia subgroups was stratified according to FAB classification, ethnicity, gender, age, FLT3 mutation status (UALCAN) and PML/RAR-fusion status.** (**A**) CASP1 mRNA expression with different FAB classification was shown in leukemia. (**B**) CASP1 mRNA expression with different ethnicity was shown in leukemia. (**C**–**F**) Boxplot showed relative expression of CASP1 in LAML in gender, age, FLTS mutation status, PML/RAR-fusion, respectively. Using t-test to assess the differences in gene expression levels between groups.

Using the Oncomine dataset, we discovered that CASP1 ranked in the top 15% based on mRNA expression levels in the Coustain-Smith Leukemia and Haslinger Leukemia datasets (Figure 2A–2C). The DNA copy number levels of CASP1 were higher in leukemia than in normal blood samples (Figure 2D), while significance difference was not observed. To further analyze the correlation between CASP1 expression and clinicopathological features, we conducted a subgroup analysis by UALCAN database. As shown in Figure 3, an increased CASP1 was found in AML patients with M3/M4/ M5 subtype, Caucasian ethnicity (Figure 3 and Tables 1, 2, *P*<0.05). Thus, the expression of CASP1 may be a promising diagnostic indicator for leukemia.

**Table 1:** The statistics of CASP1 expression in AML based on French American British classification.

**Comparison**	**Statistical significance**
M0-vs-M3	1.783800E-03
M0-vs-M5	1.541710E-02
M1-vs-M3	1.29310000485106E-08
M1-vs-M4	9.413900E-04
M1-vs-M5	5.020500E-03
M2-vs-M3	3.84020004684515E-09
M2-vs-M4	4.358400E-04
M2-vs-M5	3.716800E-03
M3-vs-M4	7.68829999664433E-08
M3-vs-M5	1.826020E-04

**Table 2:** The statistics of CASP1 expression in AML based on patients’ race.

**Comparison**	**Statistical significance**
Caucasian vs. African–American	1.556010E-02

### CASP1 is upregulated in AML cell lines and patients

**Figure 4:**
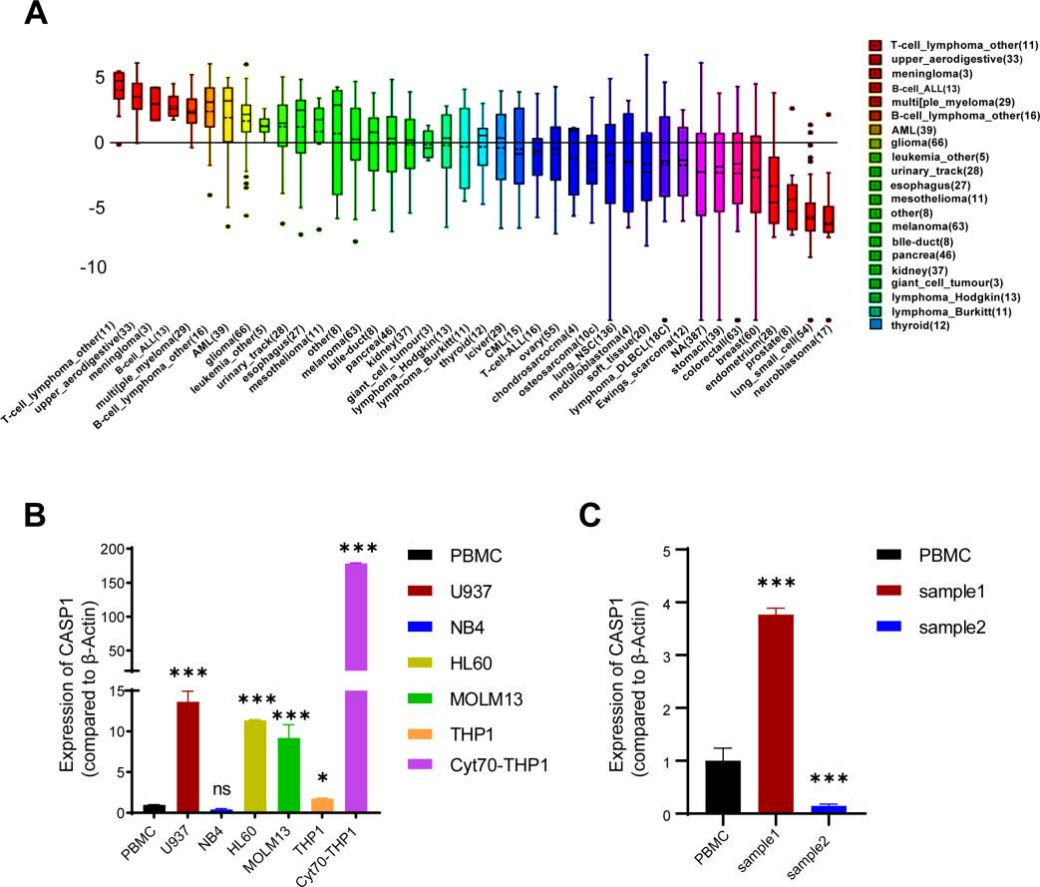
**CASP1 expression in leukemia cell lines and AML patients.** (**A**) CASP1 expression in multiple cancer cell lines (CCLE). (**B**) CASP1 expression in AML cell lines and PBMC. (**C**) CASP1 expression of 2 patients at initial relapse of AML (sample 1) and at newly diagnosed of AML (sample 2). *, p < 0.05; **, p < 0.01; ***, p < 0.001.

We inquired the CCLE database to analyse the CASP1 expression in multiple cell lines and discovered a higher transcriptional expression of CASP1 in leukemia cell lines compared to other cancer types (Figure 4A). We then performed quantitative real-time PCR assay to examine the CASP1 expression in several AML cell lines and peripheral blood mononuclear cells (PBMCs). As expected, the expression of CASP1 showed higher in most AML cell lines than in PBMCs (Figure 4B). Interestingly, we found that the CASP1 expression was dramatically higher in MLL-rearranged AML cell lines and cytarabine-resistant cell (Figure 4B and Supplementary Figure 1). Further analysis of AML patients showed that CASP1 expression was higher in initial relapse of AML (Sample 1) than in newly diagnosed of AML (Sample 2) (Figure 4C). In sum, the expression of CASP1 was consistent with that of database.

### Evaluating the prognostic value of CASP1 in AML

**Figure 5:**
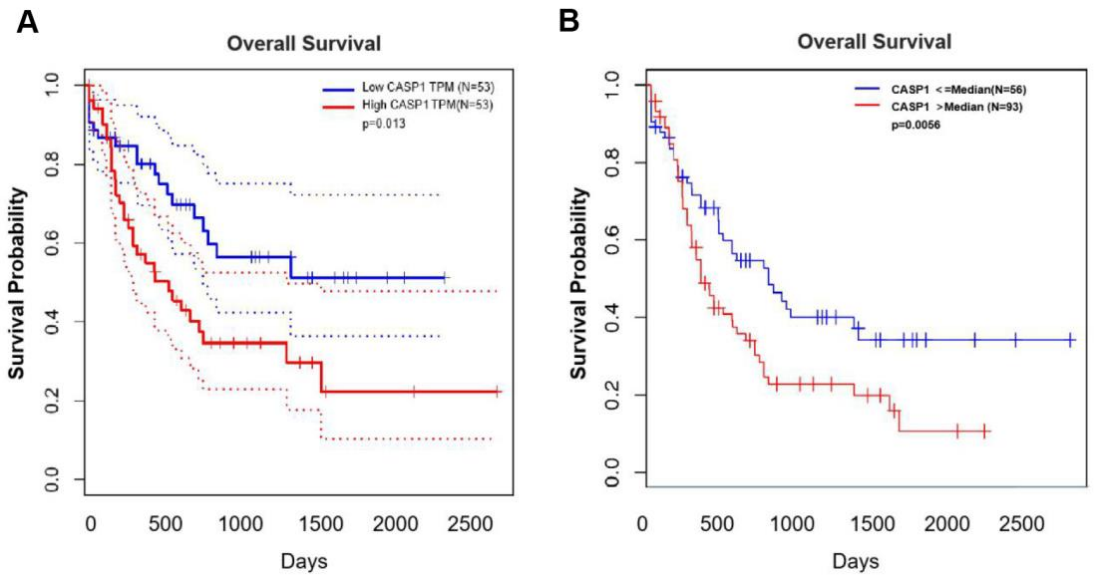
**The prognostic value of mRNA level of CASP1 in AML (GEPIA and LinkedOmics).** (**A**) GEPIA showed the prognostic value of CASP1 mRNA expression levels in AML. (**B**) LinkedOmics showed the prognostic value of CASP1 mRNA expression levels in AML.

We evaluated the relationship between CASP1 expression and prognosis information in AML through the Kaplan-Meier survival curves (Figure 5). Patients were grouped into low-CASP1 and high-CASP1 expression group based on the median value of CASP1 expression. The survival analysis revealed that patients with elevated CASP1 expression had a shorter OS than those with low levels of CASP1 expression (Figure 5A, *P* < 0.05). Similarly, in LinkedOmics, increased CASP1 was associated with poor OS in AML (Figure 5B, *P* < 0.05). Therefore, these results indicated that CASP1 may be a factor for predicting the prognosis of AML patients.

**Figure 6:**
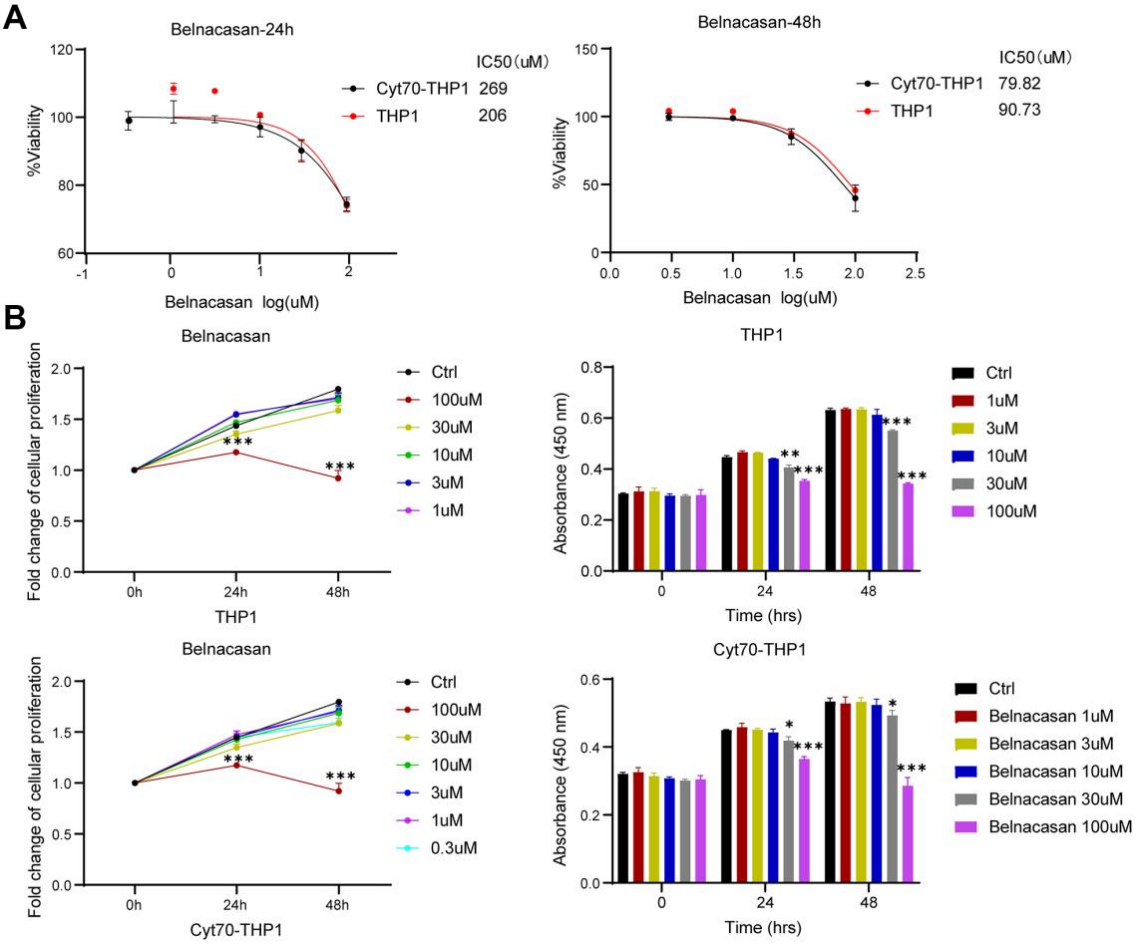
**Inhibition of CASP1 impairs the proliferation of THP1 and Cyt70-THP1.** (**A**) CCK-8 assay was used to investigate the IC50 of THP1 and Cyt70-THP1 after CASP1 inhibition for 24 and 48h. (**B**) CCK-8 assay was used to investigate the proliferation of THP1 and Cyt70-THP1 after CASP1 inhibition for 24 and 48h. * p < 0.05, ** p < 0.01, ***p < 0.0005.

Since CASP1 is significantly overexpressed in AML and Cytarabine-resistant cells, and is associated with poor prognosis, we inhibited CASP1 with Caspase-1 inhibitor Belnacasan to investigate its role. We evaluated the effect of CASP1 on proliferation by using CCK-8 assay. We found that CASP1 inhibition reduced the IC50 and proliferation of THP1 and Cyt70-THP1 in a time-dependent manner (Figure 6A, 6B). And Cyt70-THP1 seemed to show a more pronounced decrease in IC50 than THP1 (Figure 6A, 6B). These suggested that CASP1 inhibition could impair the proliferation of AML cells.

### CASP1 co-expression networks in leukemia

**Figure 7:**
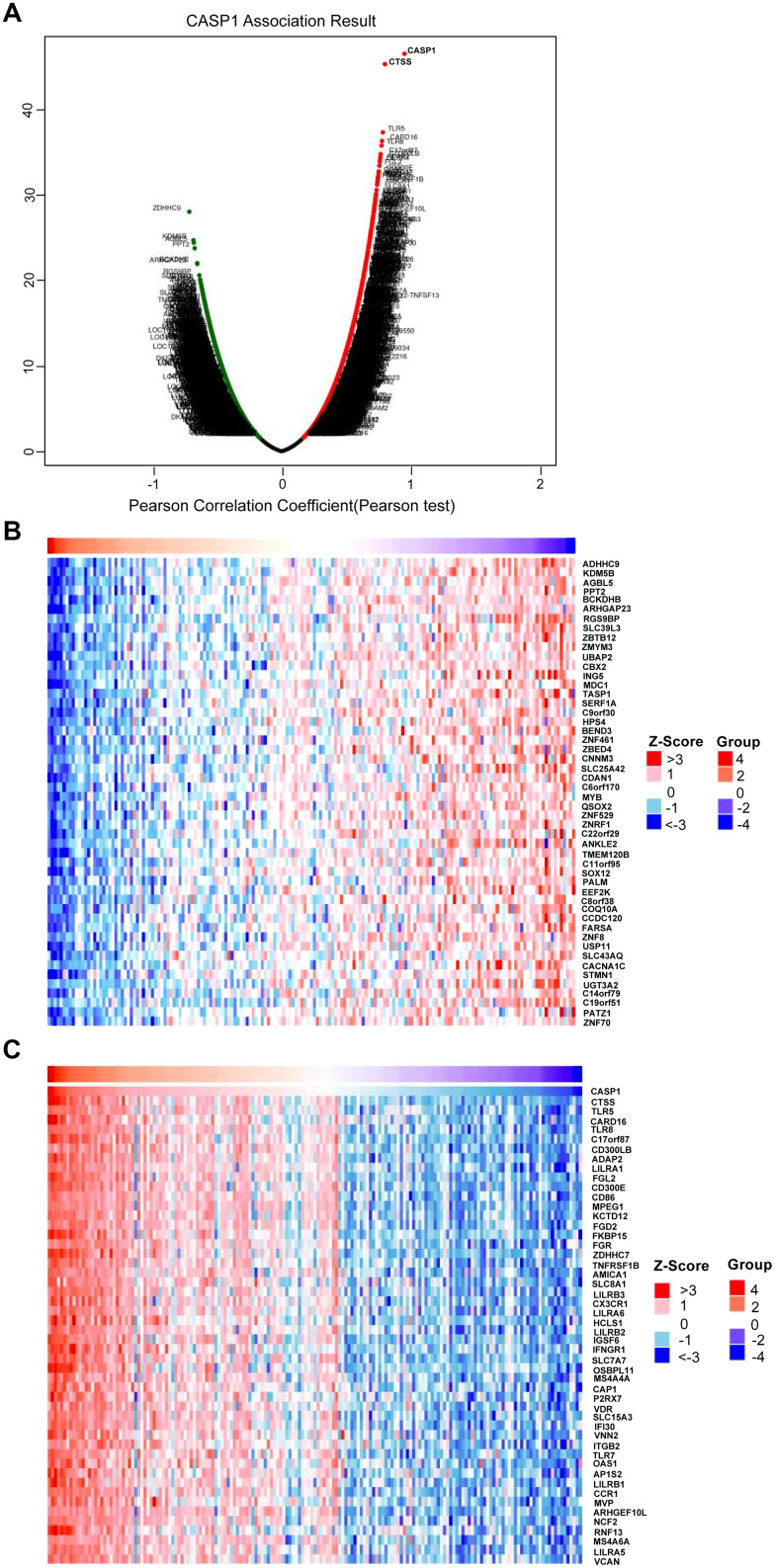
**Genes differentially expressed in correlation with CASP1 in AML (LinkedOmics).** (**A**) Using Pearson test to analyze genes differentially expressed in correlation with CASP1 in AML. (**B**, **C**) Heat maps showed that top 50 genes were significantly negatively and positively correlated with CASP1, respectively.

We used the LinkedOmics function module to analyze co-expression genes of CASP1 for exploring its biological meaning in AML. Figure 7A showed that 2,600 and 4,666 genes were significantly positively and negatively correlated with CASP1, respectively (false discovery rate, FDR < 0.01). Furthermore, Figure 7B, 7C showed that top 50 genes were significantly negatively and positively correlated with CASP1, respectively. There was a strong positively correlation between CASP1 and CTSS (Pearson correlation = 0.83, P = 8.427E-47), TLR5 (Pearson correlation = 0.79, P = 3.134e-38), CARD16 (Pearson correlation = 0.78, P = 3.271E-37), which reflect inflammatory and immune response. The correction scatter diagrams of the top 3 significant genes correlated with CASP1 are shown in Supplementary Figure 2.

**Figure 8:**
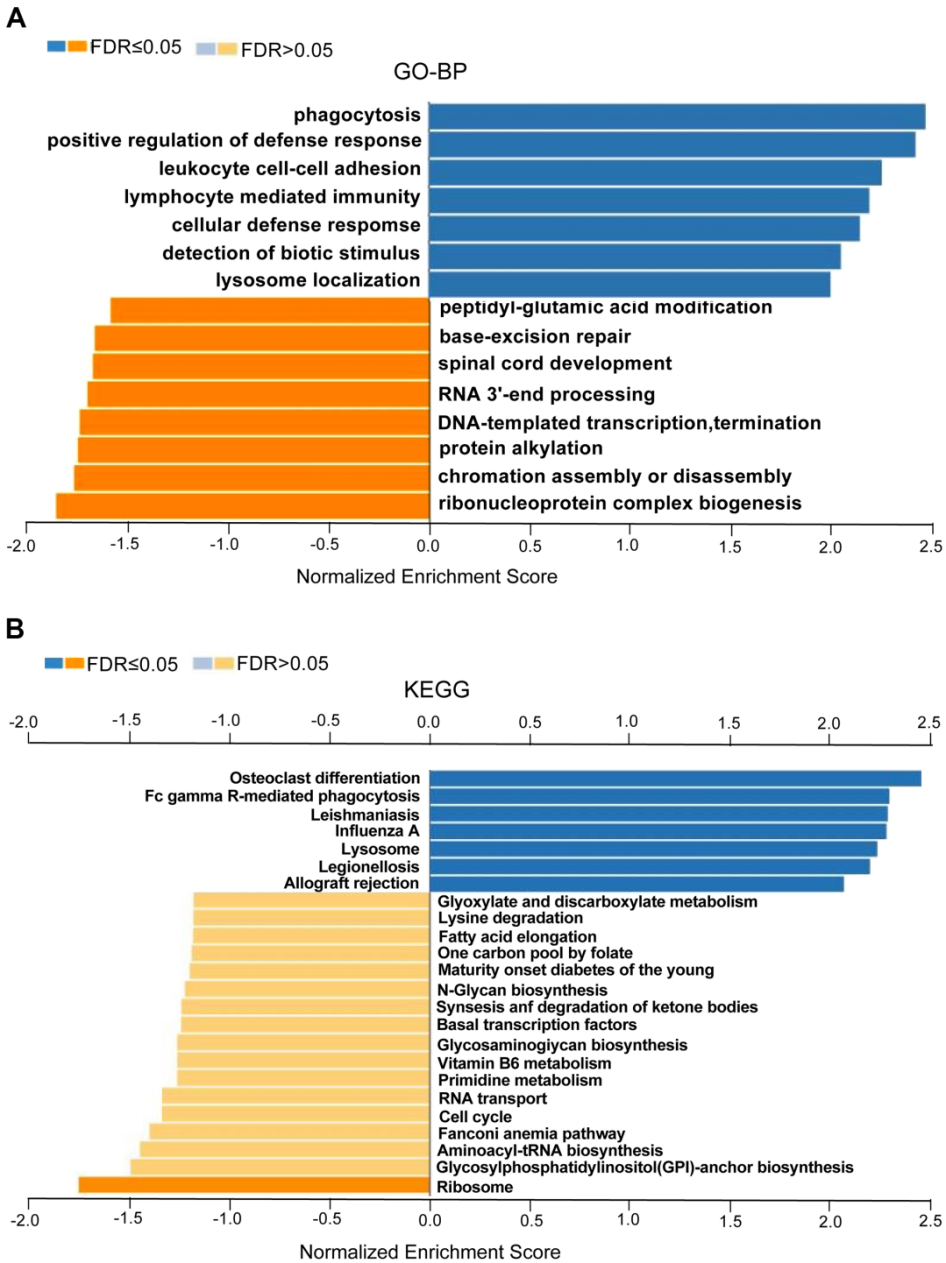
**GO annotations and KEGG pathways enriched with CASP1 co-expressed genes in leukemia (LinkedOmics).** Using GSEA to analyze the significantly enriched GO_BP and KEGG pathways of CASP1 co-expression genes in LAML. (**A**) Gene Ontology_Biological processes, analyzed by GSEA. (**B**) KEGG pathway, analyzed by GSEA. The LeadingEdgeNum and the false discovery rate (FDR) are shown in blue and orange column, respectively.

We found that these co-expression genes associated with CASP1 were involved primarily in phagocytosis, interleukin-1 production, lymphocyte mediated immunity, cellular defense response and leukocyte, while activities base excision repair, RNA 3'-end processing, spinal cord development, protein alkylation, DNA-templated transcription, termination, chromatin assembly or disassembly and ribonucleoprotein complex biogenesis were strongly inhibited through Gene Ontology (GO) term analysis (Figure 8A). In addition, we performed Kyoto Encyclopedia of Genes and Genomes (KEGG) analysis, confirmed that co-expression gene of CASP1 enrichment in leishmaniasis, lysosome, legionellosis, endocytosis, cytokine receptor interaction, NOD-like, and B cell receptor signaling pathway (Figure 8B). In summary, CASP1 has widespread impact on immune response in leukemia.

### Kinase, miRNA and transcription factor of CASP1 networks in AML

Here we used LinkedOmics to further explore the regulators of CASP1 in AML, in which we analyzed the enrichment in kinases, miRNAs and transcription factors (TF) of CASP1 positively related genes. Lck/yes-related novel protein tyrosine kinase (LYN) and lymphocyte-specific protein tyrosine kinase (LCK) are significant kinases primarily associated with positive genes (Table 3). In addition, mutated and overexpressed LCK is driving leukemia cell proliferation [19]. LYN kinase was significantly associated with the OS of AML [20]. There are no significantly enriched miRNA for CASP1 coexpressed genes (Table 3 and Supplementary Table 1). The E2F transcription factor family, including V$E2F_01, V$E2F1_Q6_01, V$E2F_Q6_01, V$E2F1_Q4_01, V$E2F_Q3_01, V$E2F_03, V$E2F_Q4_01 and V$E2F_Q4_01, is significant TF correlated with positive genes (Table 3 and Supplementary Table 2). The transcription factor E2F family is identified as an oncogene or antioncogene. The properties of these regulators in AML may give us insight into the pathogenesis of leukemia [21]. It has become clear that E2F1 expression can regulate the prognosis of leukemia [22]. Pellicano et al. [23] shown that E2F1 plays a crucial regulatory role in the proliferation state of chronic myeloid leukemia (CML) stem/progenitor cells (SPC).

**Table 3:** The kinase, miRNA and transcription factor of CASP1 networks in AML.

**Enriched category**	**Geneset**	**LeadingEdgeNum**	**FDR**
Kinase Target	Kinase_LYN Kinase_LCK	19 16	0.0077359 0.023208
miRNA Target	GGCCAGT,MIR-193A,MIR-193B	19	0.27124
	GGGATGC,MIR-324-5P	13	0.38149
	CAGTCAC,MIR-134	15	0.47766
	TCTAGAG,MIR-517	12	0.49457
Transcription Factor	V$SRF_01	16	0.0024893
	V$IRF_Q6	84	0.0026671
	RGAGGAARY_V$PU1_Q6	94	0.0072739
	V$E2F1_Q4_01	77	0.0082841
	V$CEBP_Q2_01	50	0.040133
	V$ETS_Q4	47	0.027004
	V$MAF_Q6	50	0.035935
	V$E2F1DP1_01	82	0.049704
	WCTCNATGGY_UNKNOWN	28	0.047219
	GGAMTNNNNNTCCY_UNKNOWN	36	0.04869

## DISCUSSION

CASP1 is a member of the cysteine-aspartic acid protease (caspase) family. Cascade activation of caspases is closely related to apoptosis processes [24]. However, it is inconsistent with the results of the expression of CASP1 in prostate cancer and ovarian cancer [15, 16]. Meanwhile, CASP1 and its activator NLRP3 are the core component of the inflammasome, and its overexpression in leukemia leads to glucocorticoid resistance [18]. Furthermore, genetic variation of CASP1 is associated with risk of chronic lymphocytic leukemia [25]. We aimed to discover the mRNA expression and prognostic values of CASP1 in AML. We anticipated that these results would be useful for improving current treatment regimens and outcomes of AML. Therefore, we performed a detailed analysis of cancer versus normal samples to explore the relationship between CASP1 and AML.

First, we found that mRNA expression levels and CNVs of CASP1 in AML were higher than in normal blood samples, analyzed by ONCOMINE and GEPIA databases (Figures 1, 2). The mRNA of CASP1 was also overexpressed in leukemia cell lines, analyzed by CCLE (Figure 4A). Then, we verified the mRNA expression of CASP1 in THP1, MOLM13, HL60, U937, NB4 and PBMCs, respectively. We found that the expression of CASP1 in most cell lines was higher than PBMCs (Figure 4B). THP1 and MOLM13 cell lines harbor a t (9;11) translocation resulting in the MLL-AF9 fusion gene. U937 harbors a t (10;11) (p13; q14) translocation resulting in the CALM-AF10 fusion gene. Standard chemotherapeutic strategies are often ineffective in treating patients with MLL-AF9 and CALM-AF10 fusions [26]. CASP1 expression of 2 patients at initial relapse of AML (Sample 1) and at newly diagnosed of AML (Sample 2). We observed significantly higher expression of CASP1 in relapsed AML patient. Therefore, we speculated that CASP1 was highly expressed in most leukemia patients, especially in patients with poor prognosis. Furthermore, overexpression of CASP1 was strongly related to poor survival in multiple leukemia cohorts (Figure 5). Therefore, our results indicated that CASP1 is overexpressed in multiple leukemia and is a potent prognostic indicator, particularly for AML. We will further confirm it in subsequent experiments.

Second, we conducted an analysis of the clinic-pathological features of AML samples from the UALCAN database to further understand the relationship between CASP1 and clinic-pathological features. As shown in Figure 3, an increased CASP1 was found in AML patients with M3/M4/ M5 subtype, Caucasian ethnicity (Figure 3 and Tables 1, 2, *P*<0.05). Thus, the expression of CASP1 may be a promising diagnostic indicator for leukemia.

Third, we evaluated the relationship between CASP1 expression and prognosis information in AML through the Kaplan-Meier survival curves (Figure 5). The survival analysis revealed that patients with elevated CASP1 expression had a shorter OS than those with low levels of CASP1 expression (Figure 5A, 5B, *P* < 0.05). Since CASP1 is significantly overexpressed in AML and Cytarabine-resistant cells, and is associated with poor prognosis, we inhibited CASP1 with Caspase-1 specific inhibitor Belnacasan to investigate its role. We evaluated the effect of CASP1 on proliferation by using CCK-8 assay and found that CASP1 inhibition reduced the IC50 and proliferation of THP1 and Cyt70-THP1 in a time-dependent manner (Figure 6A, 6B). And Cyt70-THP1 seemed to show a more pronounced decrease in IC50 than THP1 (Figure 6A). But the IC50 of Belnacasan is relatively high, considering that the drug may affect the survival of leukemia cells through inflammatory or immune microenvironment. These suggested that CASP1 inhibition could impair the proliferation of AML cells. Therefore, these results indicated that CASP1 may be a factor for predicting the prognosis and a therapeutic target of AML patients.

Fourth, the LinkedOmics function module was used to analyze CASP1 co-expression genes to further explore the biological meaning of CASP1 in AML. Related functional networks were involved in phagocytosis, lymphocyte mediated immunity, cellular defense response, cytokine receptor interaction, NOD-like receptor, and B cell receptor signaling pathway. These results demonstrated that CASP1 overexpression had widespread impact on inflammatory response and immune response (Figure 8A, 8B), and was involved in the pathogenesis of AML [25].

Finally, we assessed regulators of CASP1 in AML by analyzing the enrichment of kinases, miRNAs, and transcription factor (TF) of CASP1 positively related genes. We discovered that CASP1 is primarily related to LYN kinase and LCK kinase in leukemia (Table 3). Mutated and overexpressed LCK drive leukemia cell proliferation [19]. Moreover, LYN kinases were highly expressed in AML and associated with poor prognosis [20]. The myeloid Src-kinase Lyn family had been reported as a target for AML drug therapy, and inhibition of LYN was a potent strategy for AML treatment [27]. Next, the E2F family was the main transcription factor in abnormal regulation of CASP1 (Table 3 and Supplementary Table 2). Studies showed that E2F family was a powerful regulator in cell cycle progression [28]. In addition, it had been clear that E2F family regulates DNA replication, DNA repair, differentiation and cell proliferation [28, 29]. Consequently, the transcription factor E2F family is regarded as an oncogene or antioncogene [30]. The properties of these regulators in AML may give us insight into the pathogenesis of leukemia [21]. There was a similarly strong correlation between High E2F1 expression and poor prognosis of acute leukemia (AL) [22]. Pellicano et al. [23] showed that E2F1 played a crucial regulatory role in the proliferation state of Chronic myeloid leukemia (CML) Stem progenitor cells (SPC) [23]. Our study indicated that E2F1 may be a crucial factor of CASP1 in regulating the proliferation of leukemia cells, and was consistent with previous reports [29, 30]. However, no miRNAs significantly associated with CASP1 were found. For this study, online tools based on bioinformatics theory were used for target gene analysis of tumors data in public databases. Large sample size, low cost, and strong operability are the advantages of this method.

The results indicated that the increased expression of CASP1 may be a molecular marker for the high-risk subgroup of AML. In addition, the up-regulation of CASP1 in AML may have far-reaching effects on cell proliferation, inflammatory and immune response. In short, our research demonstrates the importance of CASP1 in AML and recommends its use as a potential prognostic and therapeutic target. This approach allows us to explore a wider range of potential targets and accelerate the clinical transformation of drugs. At the same time, our research has their own limitations. We have no large samples and animal experiments to support the above results. But these problems will be further solved in subsequent experiments.

## MATERIALS AND METHODS

### Cell culture

The AML cell lines THP1, MOLM13, HL60, U937 and NB4 were grown in RPMI-1640 medium (Gibco) containing 10% fetal bovine serum (FBS), 2 mM glutamine, 100μg/ml penicillin, and streptomycin (Sigma) at 37° C with 5% CO2. Cells were passaged every 2–3 days.

### Cytarabine selection

Multiple step selections with gradually increasing Cytarabine concentrations (cat. 147-94-4, Huateng) was performed on THP1 cells for the establishment of drug-resistant sublines, using a starting dose of approximately its original IC30 (1uM, 72hours) values; the latter were obtained by growth inhibition assays as detailed below. THP1 cells were continuously grown in 1uM Cytarabine for 28 days until cells resumed their original doubling time, yielding a drug-resistant subline termed Cyt30-THP1 (THP1 Ara-C IC30 resistant); at this passage (day 28 from initiation of drug selection), Cyt30-THP1 cells were frozen down in aliquots and thawed for any experiment that required the original cells. Cyt30-THP1 cells were also transferred into 1.6μM (IC50) Cytarabine, resulting in the sunlines Cyt50-THP1. Cyt50-THP1 cells were also transferred to grow in either 2.7μM (IC70) Cytarabine, resulting in the sunlines Cyt70-THP1. Following their establishment, Cyt70-THP1 cells were also grown in drug-free medium to evaluate the stability of their drug resistance phenotype.

### Patient samples

Two AML patients who presented to the Seventh Affiliated Hospital of Sun Yat-Sen University were included in this study. Diagnosis of AML was based on morphology, immunophenotype, cytogenetics and molecular analysis (MICM). In addition, one healthy volunteer in whom cancers were ruled out by a comprehensive medical examination were enrolled as normal controls. Mononuclear cells were isolated from peripheral whole blood of AML patients and volunteers using density gradient centrifugation and then dispersed in Trizol reagent (NucleoZol, Germany) and cells were stored at −80° C for further analysis. Informed consent was obtained from all the participants and the study was approved by the Ethics Committee of the Seventh Affiliated Hospital of Sun Yat-Sen University.

### Growth inhibition assays

Belnacasan (VX-765, cat. T6090), was purchased from TOPSCIENCE. For the analysis of cell lines, THP1 cells were grown in RPMI-1640 medium. Cyt70-THP1 cells continuously growing in Cytarabine-containing medium were grown in drug-free medium for 3 days prior to experiments. THP1 and Cyt70-THP1 cells were seeded into a 96-well plate (1×10^4^ cells/well) and cultured in RPMI 1640 medium at 37° C for 24 h. Then, the cells were treated with various concentrations of Belnacasan for 48 h. Cells treated with DMSO instead of Belnacasan served as the control group. Finally, cell viability was detected using a CCK-8 kit according to the manufacturer’s instructions. The experiment was performed at least 3 times.

### QRT-PCR

TRIzol reagent (NucleoZol, Germany) was used to extract total RNA from the AML cells. β-actin was used as an internal control. cDNAs were generated using the HiScript II Q RT reagent kit (Vazyme) in line with the manufacturer’s instructions. ChamQ Universal SYBR qPCR Master Mix (Vazyme) was used to analyze the synthesized cDNAs according to the manufacturer’s instructions. Primer sequences used for real-time PCR were:

CASP1: Forward: 5′GCCTGCCGTGGTGATAATGT3′;

Reverse: 3′TCACTCTTTCAGTGGTGGGC5′.

β-actin: Forward: 5′CCTGTACGCCAACACAGTGC3′;

Reverse: 3′ATACTCCTGCTTGCTGATCC5′.

### Statistical analyses

All data are displayed as the mean ± SEM. GraphPad Prism 8 statistical software was used for statistical analyses. Comparisons between groups were performed using the t test or ANOVA. *p*<0.05 was considered as statistically significant.

### Database descriptions

### GEPIA database analysis

GEPIA (Gene Expression Profiling Interactive Analysis) is an online database including 8,587 normal samples and 9,736 tumors from TCGA and Genotype-Tissue Expression (GTEx) projects [31]. We performed differential gene expression analysis and survival analysis between tumors with normal samples. It can also be used to perform similar gene detection, correlation analysis, and Principal Component Analysis [31]. The *p* value was used to assess the significance of the difference, and *p* < 0.05 was considered to be significant.

### Oncomine database analysis

Oncomine (https://www.oncomine.org/) is an online cancer sample database that was used to analyze CASP1 mRNA expression and DNA copy number. This analysis drew on a series of leukemia studies, including Coustan-Smith Leukemia, Haslinger Leukemia, and TCGA Leukemia 2 [32, 33]. We performed differential gene expression and DNA copy number analysis between tumors with normal samples. The *p* value and fold change were used to assess the significance of the difference, and *p* < 0.01 and fold change >1.5 was significant.

### UALCAN database analysis

UALCAN (http://ualcan.path.uab.edu) is an interactive web-portal including clinical data from 31 cancer types and TCGA level 3 RNA-seq [34]. It can be used to analyze differential gene expression and multiple clinicopathological features between tumors with normal samples. We conducted a subgroup analysis of various clinic-pathological characteristics of AML patients samples from the UALCAN.

### CCLE database analysis

The CCLE is a project including of gene expression, chromosomal copy number, and DNA mutations from 947 human cancer cell lines covering more than 30 tissues [35]. This database allows us to explore the expression of CASP1 in different cancer types. CCLE is publicly available at https://www.broadinstitute.org/ccle.

### LinkedOmics database analysis

The LinkedOmics (http://www.linkedomics.org/login.php) is an interactive web-portal including 32 TCGA cancer-associated data [36]. Using Pearson test to analyze genes differentially expressed in correlation with CASP1 in AML, shown in heat maps. We used the LinkedOmics function module to analyze co-expression genes of CASP1 for exploring its biological meaning in leukemia. Using GSEA, we performed analysis of GO_BP, KEGG pathways and target enrichment of kinase, miRNA as well as transcription factor. The threshold was determined based on the following values: FDR of 0.05, simulations of 500. In addition, we evaluated its prognosis value in leukemia by clinical analysis. The p value was used to assess the significance of the difference, and *p* < 0.05 was considered to be significant.

### Database statistical analysis

We performed differential gene expression and DNA copy number analysis between tumors with normal samples by using the t-test. The p value and fold change were used to assess the statistical significance of the difference, and *p* < 0.01 and fold change >1.5 was considered to be significant. We also used t-test to conduct a subgroup analysis of various clinic-pathological characteristics of AML patients’ samples from the UALCAN, and *p* < 0.05 indicates the significance of difference. We used Kaplan-Meier curves to compare the prognosis of AML patients with different median expression levels of CASP1. The log-rank test *p* < 0.05 indicates that the prognosis analysis is statistically significant. We calculated the correlation between CASP1 and CTSS, TLR5, and CARD16 gene expression levels using the Pearson correlation coefficient. The threshold of correlation was determined based on the following values: *p* of 0.05.

## Supplementary Material

Supplementary Figures

Supplementary Tables
