# Supplementary material for: Expression and prognosis analyses of CASP1 in acute myeloid leukemia
Source: Aging (Albany NY). 2021 May 17;13(10):14088–108. doi: 10.18632/aging.203028 (PMC8202835; doi:10.18632/aging.203028)
Supplement: Supplementary Figures [file aging-13-203028-s001.pdf]

SUPPLEMENTARY FIGURES

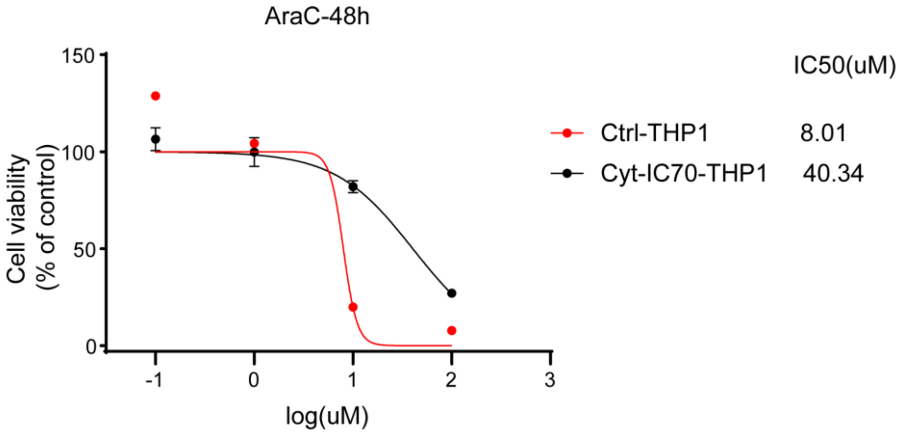

Supplementary Figure 1. Cytarabine dose-dependent growth inhibition of THP1 cells and their subline Cyt-IC70-THP1.

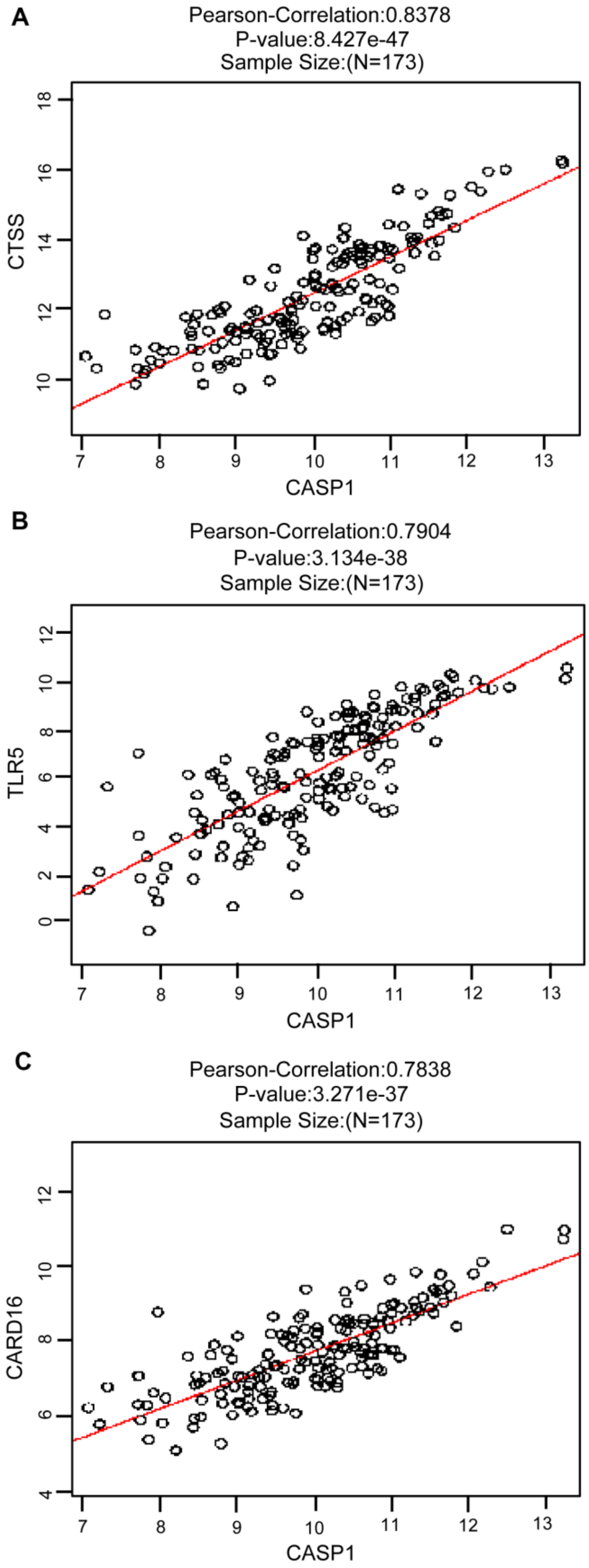

Supplementary Figure 2. The correction between CTSS, TLR5, CARD16 with CASP1 in leukemia, analyzed by LinkedOmics. (**A**) The correction between CTSS with CASP1 in leukemia. (**B**) The correction between TLR5 with CASP1 in leukemia. (**C**) The correction between CARD16 with CASP1 in leukemia.
