# Supplementary material for: Expression and prognosis analyses of CASP1 in acute myeloid leukemia
Source: Aging (Albany NY). 2021 May 17;13(10):14088–108. doi: 10.18632/aging.203028 (PMC8202835; doi:10.18632/aging.203028)
Supplement: Supplementary Tables [file aging-13-203028-s002.pdf]

SUPPLEMENTARY TABLES

Supplementary Table 1. miRNA enrichment of CASP1 co-expressed genes.

Geneset	Enrichment score	Normalized enrichment score	pvalue	FDR	Size	Leading EdgeNum
CAGTCAC,MIR-134	-0.5356147	-1.5057118	0.012397	0.307969	46	15
GGGATGC,MIR-324-5P	-0.5395772	-1.5411925	0.004405	0.308328	46	13
CGCTGCT,MIR-503	-0.5996193	-1.4712781	0.041667	0.322831	22	13
TGCACGA,MIR-517A,MIR-517C	-0.6180279	-1.4177299	0.044898	0.438749	16	3
TCTAGAG,MIR-517	-0.4748998	-1.3425616	0.075556	0.535813	45	12
GGTAACC,MIR-409-5P	-0.5314163	-1.3730561	0.069767	0.547899	27	5
GGCCAGT,MIR-193A,MIR-193B	-0.4799717	-1.5475977	0.004926	0.575831	83	19
AGTCAGC,MIR-345	-0.4490602	-1.3434165	0.06278	0.608981	54	17
GTGGTGA,MIR-197	-0.379348	-1.1863224	0.156951	0.629742	67	18
CCTGCTG,MIR-214	-0.3322438	-1.1932098	0.055249	0.629751	220	44
CAGCCTC,MIR-485-5P	-0.3823391	-1.2927548	0.028846	0.639001	131	35
ATGTTTC,MIR-494	-0.3411439	-1.1966772	0.089474	0.646199	141	36
GGCGGCA,MIR-371	-0.7508409	-1.3047399	0.136531	0.649839	5	3
CAGGGTC,MIR-504	-0.3825697	-1.1988028	0.116279	0.671615	78	21
AGGGCCA,MIR-328	-0.4041804	-1.2586383	0.09375	0.684516	73	24
GTGTCAA,MIR-514	-0.4130576	-1.2703542	0.093617	0.68502	59	16
CCCAGAG,MIR-326	-0.3471427	-1.2023533	0.09596	0.690066	141	25
TCCCCAC,MIR-491	-0.4121849	-1.2101717	0.132159	0.692994	55	15
CAGCACT,MIR-512-3P	-0.3292829	-1.135226	0.15311	0.700912	143	43
TCGATGG,MIR-213	-0.6772519	-1.1432002	0.310714	0.712765	5	4
AATGGAG,MIR-136	-0.3630656	-1.13698	0.181818	0.718277	74	18
GTTTGTT,MIR-495	-0.3044444	-1.1160896	0.147541	0.719442	235	69
AGGAGTG,MIR-483	-0.4095191	-1.2127934	0.120773	0.720192	61	16
CAGCAGG,MIR-370	-0.3218814	-1.1096571	0.176768	0.722945	137	17
ACAGGGT,MIR-10A,MIR-10B	-0.3334561	-1.1202692	0.190217	0.724158	119	20
AAGCCAT,MIR-135A,MIR-135B	0.33712366	1.19216267	0.065672	0.818531	310	43
GTGACTT,MIR-224	0.36301753	1.19240037	0.130872	0.857659	150	31
CTTTGCA,MIR-527	0.32108688	1.082235	0.254386	0.86316	221	29
ATTCTTT,MIR-186	0.31759521	1.09460462	0.248387	0.86323	251	49
GTCTACC,MIR-379	0.51560807	1.19760904	0.206349	0.871802	20	3
CTCCAAG,MIR-432	0.3607626	1.06810144	0.29588	0.874074	74	16
GGATCCG,MIR-127	0.58680985	1.21240622	0.231441	0.875448	10	3
AATGTGA,MIR-23A,MIR-23B	0.30464776	1.08335814	0.25731	0.878253	388	64
TAATAAT,MIR-126	0.32201754	1.08721064	0.269841	0.878776	207	34
TTTGCAG,MIR-518A-2	0.32615264	1.0958745	0.231013	0.879395	193	33
ATGTTAA,MIR-302C	0.35198092	1.20279444	0.09009	0.887306	228	35
GTGCCAA,MIR-96	0.30565774	1.06837271	0.29582	0.892419	283	53
TACTTGA,MIR-26A,MIR-26B	0.31273251	1.09627075	0.215873	0.901719	285	55
CTCAGGG,MIR-125B,MIR-125A	0.34406849	1.21625446	0.058282	0.904954	303	89
TCTGGAC,MIR-198	0.33218434	1.0140016	0.409894	0.906819	82	16
ACCAAAG,MIR-9	0.29584667	1.0691252	0.240331	0.910421	458	57
CAGTATT,MIR-200B,MIR-200C,MIR-429	0.29035348	1.05676528	0.261838	0.912723	440	69
CTGAGCC,MIR-24	0.29625819	1.00895049	0.460064	0.913255	219	31

GTGCAAT,MIR-25,MIR-32,MIR-92,MIR-363,MIR-367	0.28211347	0.99537175	0.461538	0.915301	294	50
GTGCCTT,MIR-506	0.31351677	1.15712834	0.060526	0.917579	674	144
TTTTGAG,MIR-373	0.32583634	1.1026909	0.233846	0.918391	210	28
AGCATTA,MIR-155	0.35282527	1.11720312	0.244068	0.920096	128	29
AAGCACT,MIR-520F	0.30106978	1.01432611	0.422442	0.921389	219	44
TACAATC,MIR-508	0.41788572	1.1689215	0.234848	0.922923	57	14
ATGCAGT,MIR-217	0.32340869	0.9970131	0.468852	0.92301	103	14

Supplementary Table 2. Transcription factor enrichment of CASP1 co-expressed genes.

Geneset	Enrichment score	Normalized enrichment score	pvalue	FDR	Size	Leading EdgeNum
V$PEA3_Q6	0.57396322	1.96059442	0	0	240	64
V$E2F_01	-0.5999168	-1.8352204	0	0.002347	64	35
V$PU1_Q6	0.5411158	1.81098492	0	0.003574	208	63
V$SRF_01	0.65959571	1.80023909	0	0.003574	47	16
V$ELF1_Q6	0.55037567	1.86720217	0	0.004468	220	62
V$IRF_Q6	0.53577782	1.8177585	0	0.004765	228	84
V$ISRE_01	0.50728611	1.74621947	0	0.005659	233	76
RYTTCCTG_V$ETS2_B	0.45066767	1.72071227	0	0.007914	1002	222
V$TEL2_Q6	0.49657672	1.70003045	0	0.010276	220	54
STTTCRNTTT_V$IRF_Q6	0.51339992	1.69480119	0	0.010722	175	48
RGAGGAARY_V$PU1_Q6	0.46921073	1.67433248	0	0.011765	459	94
YNTTTNNNANGCARM_UNKNOWN	0.58236407	1.68595935	0	0.011795	67	10
V$IRF1_Q6	0.49473696	1.67451422	0	0.012834	233	55
V$E2F1_Q6_01	-0.4839508	-1.7537136	0	0.014084	218	91
V$ETS2_B	0.47904718	1.65316635	0	0.014159	259	55
V$ICSBP_Q6	0.47956267	1.62511362	0	0.019658	230	62
V$E2F_Q6_01	-0.4632908	-1.6752839	0	0.022534	218	66
V$E2F1_Q4_01	-0.4690197	-1.6876398	0	0.022887	210	77
V$CEBPB_01	0.47218528	1.60792237	0	0.02347	240	54
V$ETS_Q4	0.46520902	1.59344716	0	0.028369	236	47
V$CEBPB_02	0.45616718	1.58251459	0	0.028791	235	50
V$E2F_Q3_01	-0.4701195	-1.6921283	0	0.02895	215	78
V$AP1_Q4_01	0.46035093	1.58408106	0	0.030065	243	58
ACTAYRNNNCCCR_UNKNOWN	-0.4214518	-1.6043698	0	0.035503	408	121
V$CEBP_Q2_01	0.45789441	1.56208554	0	0.035647	247	51
V$E2F_03	-0.449346	-1.6225953	0	0.038731	224	68
V$E2F_Q4_01	-0.4471581	-1.6057194	0	0.039905	215	66
V$IRF7_01	0.45553697	1.54190145	0	0.040336	231	49
SGCGSSAAA_V$E2F1DP2_01	-0.4473325	-1.5812434	0	0.040426	155	71
V$MAF_Q6	0.45346044	1.54239303	0	0.042174	239	50
V$E2F1_Q6	-0.4312803	-1.5668496	0	0.043191	213	77
V$E2F1DP1_01	-0.4161494	-1.5071466	0	0.043426	218	82
V$E2F1DP2_01	-0.4161494	-1.5071466	0	0.043426	218	82
V$E2F4DP2_01	-0.4161494	-1.5071466	0	0.043426	218	82
V$E2F_Q6	-0.4276823	-1.5559191	0	0.043532	211	85
V$MYCMAX_01	-0.4205049	-1.5483221	0	0.043621	236	66
V$SRF_C	0.44992831	1.53071115	0	0.044189	197	41
V$E2F_Q4	-0.4241098	-1.5337775	0	0.045683	212	87
WCTCNATGGY_UNKNOWN	-0.4877758	-1.5119369	0.004545	0.047085	71	28
GGAMTNNNNNTCCY_UNKNOWN	-0.4533504	-1.4950502	0.009009	0.048176	104	36
V$E2F_02	-0.4157139	-1.513259	0	0.049441	218	82
V$E2F1DP1RB_01	-0.418954	-1.5160793	0	0.050702	209	76
V$ZF5_B	-0.4153122	-1.5217067	0	0.050971	225	52
V$NMYC_01	-0.4036861	-1.4760014	0	0.056656	252	69
MCAATNNNNNGCG_UNKNOWN	-0.4717761	-1.4586272	0.009569	0.062596	76	24
V$MYCMAX_B	-0.3940211	-1.4621286	0	0.063072	245	89
V$AML1_01	0.43094581	1.48271613	0.003155	0.069993	246	55

V$AML1_Q6	0.43094581	1.48271613	0.003155	0.069993	246	55
V$E2F4DP1_01	-0.3957904	-1.4443529	0	0.070608	219	84
TGANTCA_V$AP1_C	0.38701162	1.47565026	0	0.073769	1035	221
